# Transcription Factor *VmGAL4* Governs Vegetative Growth, Development, and Virulence in *Valsa mali*

**DOI:** 10.3390/jof12070511

**Published:** 2026-07-12

**Authors:** Yufei Diao, Jiayin Zhang, Rui Cheng, Xiong Xiong, Chengli Wang, Dezhen Zhang, Chengming Yu, Huixiang Liu

**Affiliations:** 1 Jia Sixie College of Agriculture, Weifang University of Science and Technology, Weifang 262700, China; f18853886856@163.com (Y.D.); zjy18661558703@outlook.com (J.Z.); 19295363560@163.com (R.C.); zhen5198@126.com (D.Z.); 2 Shandong Research Center for Forestry Harmful Biological Control Engineering and Technology, College of Plant Protection, Shandong Agricultural University, Tai’an 271018, China; wein599@163.com (C.W.); ycm2006.apple@163.com (C.Y.); 3 Mountain Tai Forest Ecosystem Research Station of State Forestry Administration, College of Forestry, Shandong Agricultural University, Tai’an 271018, China; xiongxiong5628@163.com

**Keywords:** *Valsa mali*, *VmSom1*, transcription factor, pathogenicity, osmotic stress response

## Abstract

Apple *Valsa* canker disease, caused by *Valsa mali*, is one of the most destructive diseases of apple trees in China and seriously threatens the sustainable development of the apple industry. *VmSom1* acts as a core transcription factor in the cyclic adenosine monophosphate/protein kinase A (cAMP/PKA) signaling pathway and regulates vegetative growth, development and pathogenicity of this phytopathogen. Transcriptome analysis was performed using the *VmSom1* deletion mutant and the wild-type strain sdau11-175, and a significantly differentially expressed transcription factor, *VmGAL4*, was identified. In this study, the single-gene deletion mutant Δ*VmGAL4* and the double-gene deletion mutant Δ*VmSom1*/*VmGAL4* were constructed via homologous recombination, aiming to preliminarily explore the interaction between these two genes. Sequence analysis revealed that the *VmGAL4* protein contains a conserved fungal_TF_MHR domain spanning amino acids 164 to 614. Phylogenetic analysis indicated that *VmGAL4* shares the closest phylogenetic relationship with homologs from *Cytospora schulzeri* and *Cytospora chrysosperma*. Phenotypic assays demonstrated that the *VmGAL4* deletion mutant exhibited markedly reduced mycelial growth rate and fewer pycnidia production. Additionally, the mutant displayed enhanced sensitivity to cell wall inhibitors and osmotic stress agents, along with significantly increased capacity to utilize various carbon and nitrogen sources and decreased pathogenicity compared with the wild-type strain. Compared with the single deletion mutant Δ*VmSom1*, the double mutant Δ*VmSom1*/*VmGAL4* partially rescued the growth defects and also alleviated the reduction in pathogenicity to a certain extent. Nevertheless, conidial production remained severely inhibited in the double mutant. Collectively, *VmGAL4* is involved in the regulation of vegetative growth, asexual development, cell wall integrity, osmotic stress response, carbon and nitrogen source utilization, and pathogenicity in *V. mali*.

## 1. Introduction

Apple *Valsa* canker disease, a typical woody plant canker disease caused by the ascomycete *Valsa mali*, is widely distributed in apple-producing areas of East Asia and has become one of the most destructive diseases threatening apple cultivation [1,2]. The pathogen invades apple trees through wounds, colonizes the cortical and xylem tissues, and causes bark rot, branch dieback and even whole plant death, resulting in huge economic losses to the apple industry [3]. At present, the control of apple canker is still limited by the lack of effective resistant varieties and the difficulty of chemical agent penetration, and the excavation of key functional genes regulating the pathogenicity and development of *V. mali* is the basis for developing novel and efficient disease control strategies [4,5].

Transcription factors are core regulatory proteins in eukaryotic cells that bind to specific cis-acting elements in the promoter region of target genes to activate or inhibit gene transcription, and play a pivotal role in regulating fungal growth, development, stress response and pathogenicity [6,7]. Fungal-specific Zn_2_Cys_6_ zinc finger transcription factors and C_2_H_2_ zinc finger transcription factors are the two most important families of transcription factors in phytopathogenic fungi, and their functions in regulating fungal morphological differentiation, nutrient utilization, stress adaptation and host infection have been extensively studied [8,9]. For example, the Zn_2_Cys_6_ transcription factor EBR1 in *Fusarium graminearum* regulates polar growth of hyphae and pathogenicity [10]. The C_2_H_2_ zinc finger transcription factor *CRZ1* in *Penicillium digitatum* is involved in calcium signal transduction, and its deletion leads to defects in growth, conidiation and full virulence [11]. In *P*. *marneffei*, the C_2_H_2_ zinc finger transcription factor *HgrA* is specifically expressed during hyphal growth and modulates conidial germination and apical branching [12]. However, the functional characterization of zinc finger transcription factors in *V. mali* is still in its infancy, and the regulatory network of transcription factors involved in the pathogenic process of this pathogen remains largely unclear.

Virulence refers to the inherent ability of phytomicrobes to infect host tissues, suppress plant immunity and induce disease lesions, which serves as the core trait separating virulent pathogens from avirulent microorganisms. Virulent pathogens secrete specialized signal molecules to overcome host defense, while avirulent microbes produce symbiotic signals that promote plant growth without pathogenic damage [13]. Plants interact with surrounding beneficial and pathogenic microorganisms via diverse chemical signals; beneficial microbial signals promote plant growth and trigger defense responses while pathogenic chemical signals damage plant key organelles including chloroplasts, mitochondria and peroxisomes to hinder plant development [14]. As a typical necrotrophic fungal pathogen, *Valsa mali* relies on intact virulence regulatory networks to cause destructive apple canker worldwide.

Som1, a downstream transcription factor of the cAMP/PKA signaling pathway, is a conserved regulatory protein in filamentous fungi and plays an indispensable role in regulating fungal biological processes [15,16]. In *Verticillium dahliae*, *Som1* controls microsclerotia formation, root penetration and host colonization [17]. In *Magnaporthe oryzae*, *Som1* is essential for conidial and appressorium formation, and regulates cell wall differentiation and surface hydrophobicity [18]. In *Aspergillus fumigatus*, the deletion of *SomA* (homolog of *Som1*) leads to slow mycelial growth and blocked asexual development [19]. Our previous study confirmed that *VmSom1* is a key regulator of growth, development, pathogenicity and cell wall integrity in *V. mali*, and its deletion results in severe phenotypic defects [20]. Transcriptome analysis of the *VmSom1* deletion mutant revealed a large number of differentially expressed genes, among which *VmGAL4*, a zinc finger motif transcription factor, was significantly differentially expressed, suggesting its potential involvement in the *VmSom1*-mediated regulatory network.

In this study, we identified the *VmGAL4* gene from *V. mali* genome, constructed its single deletion mutant and *VmSom1*/*VmGAL4* double deletion mutant by homologous recombination technology, and systematically analyzed the phenotypic changes in the mutants in terms of mycelial growth, conidial production, stress response, carbon and nitrogen source utilization and pathogenicity.

## 2. Materials and Methods

### 2.1. Strains and Culture Conditions

The wild-type *V. mali* strain sdau11-175, Δ*VmSom1* mutant and all constructed mutants in this study were preserved by the Research Laboratory of Forest Pathogen and Host Molecular Interactions, College of Plant Protection, Shandong Agricultural University. All strains were cultured on potato dextrose agar (PDA) medium (200 g/L potato extract, 20 g/L dextrose, 15 g/L agar) at 25 °C in the dark for routine culture. TB3 medium (3 g/L yeast extract, 3 g/L casamino acids, 200 g/L sucrose, 15 g/L agar) containing 100 μg/mL hygromycin B (HygB) or 200 μg/mL geneticin (G418) (Solarbio, Beijing, China) was used for the screening of transformants during gene deletion and complementation.

For stress response assays, PDA media supplemented with different stress factors were prepared, including osmotic stress factors (0.5 M NaCl, 0.5 M sorbitol) and cell wall and membrane interference factors (200 μg/mL CR, 400 μg/mL CFW, 0.01% SDS). For carbon and nitrogen source utilization assays, Czapek’s medium was used as the basic medium, and sucrose (carbon source) was replaced with lactose, fructose, maltose and glucose of equal mass; NaNO_3_ (nitrogen source) was replaced with peptone, urea and (NH_4_)_2_SO_4_ of equal mass to prepare media with different carbon and nitrogen sources.

### 2.2. Bioinformatic Analysis

The full-length sequence of *VmGAL4* (Accession number KUI72477.1) was downloaded from the *V. mali* genome database [21]. Conserved domains were predicted by the NCBI Conserved Domain Search Service (http://www.ncbi.nlm.nih.gov, accessed on 12 January 2026). Homologous sequences of *VmGAL4* in other fungi were obtained from the GenBank database. A phylogenetic tree was constructed by the neighbor-joining (NJ) method with MEGA 7.0 software, and the bootstrap value was set to 1000 replicates.

### 2.3. Construction of Gene Deletion and Complementation Strains

The *VmGAL4* single deletion mutant (Δ*VmGAL4*) was constructed by homologous recombination using the double-joint PCR method [22]. Specific primer pairs were designed to amplify the 1 kb upstream and downstream flanking sequences of *VmGAL4*, and the HygB resistance gene (*hph*) fragment was amplified with primer pairs HPH-F/R. The three fragments were fused by double-joint PCR to obtain the *VmGAL4* gene replacement fragment, which was transformed into the protoplasts of the wild-type strain sdau11-175 by the polyethylene glycol (PEG)-mediated method [23].

The *VmSom1*/*VmGAL4* double deletion mutant (Δ*VmSom1*/*VmGAL4*) was constructed by transforming the *VmGAL4* replacement fragment into the protoplasts of the Δ*VmSom1* mutant, and positive transformants were screened on TB3 medium containing G418 and verified by PCR.

The complementation strain (Δ*VmGAL4-C*) was constructed using the gap repair method [24]. The full-length *VmGAL4* gene including its native promoter (about 2.0 kb) was amplified and co-transformed with the XhoI-digested plasmid pFL2 into yeast strain XK1-25. The recombinant plasmid was extracted and transformed into the protoplasts of Δ*VmGAL4*. All primers used in this study are listed in Table S1.

### 2.4. Growth Rate Determination

The wild-type strain, Δ*VmGAL4*, Δ*VmSom1*, Δ*VmSom1*/*VmGAL4* and Δ*VmGAL4-C* were inoculated with 5 mm agar plugs from the edge of fresh colonies onto PDA medium and cultured at 25 °C in the dark. The colony diameter was measured by the cross method at 3 days post-inoculation (dpi), and the growth rate was calculated. The experiment was designed with three biological replicates, and each replicate was measured three times.

### 2.5. Conidial Production and Germination Assay

To determine whether *VmGAL4* is involved in conidiation, we count the number of pycnidia in the unit colony area of different strains. Conidial suspension was prepared by eluting conidia with sterile water, and the concentration was adjusted to 1 × 10^6^ conidia/mL with a hemocytometer. For conidial germination assay, 10 μL of conidial suspension was dropped onto PDA medium, cultured at 25 °C, and the germination status was observed and photographed at 0, 12, 18, 24, 30 and 36 h post-inoculation (hpi) under a light microscope (Keyence, Shanghai, China) [20]. The experiment was designed with three biological replicates, and each replicate was measured three times.

### 2.6. Stress Sensitivity Assay

Osmotic stress conditions were created by amending PDA with 0.5 M NaCl and 0.5 M sorbitol, aiming to verify the gene’s function in regulating osmotic stress. PDA containing 300 µg/mL Congo red and 200 µg/mL fluorescent brightener was used to detect the mutant’s susceptibility to cell wall stressors. Furthermore, 0.01% SDS, a cell membrane-damaging agent, was added to PDA to evaluate the mutant’s sensitivity to cell membrane stress. Colony phenotypes were recorded 3 d post-inoculation [15]. The experiment was designed with three biological replicates, and each replicate was measured three times.

### 2.7. Carbon and Nitrogen Source Utilization Assay

Czapek’s medium was used as the basal culture medium. An equivalent concentration (30 g/L) of glucose, maltose, lactose, fructose, sucrose, and no carbon was used to formulate culture media containing distinct carbon sources. An equivalent concentration (3 g/L) of peptone, (NH_4_)_2_SO_4_, NaNO_3_, urea, and no nitrogen was used to formulate culture media containing distinct nitrogen sources. The tested strains were inoculated onto Czapek’s medium with different carbon or nitrogen sources and cultured at 25 °C in the dark for 3 d [25]. The colony diameter was measured, and the growth status was recorded. The experiment was designed with three biological replicates, and each replicate was measured three times.

### 2.8. Pathogenicity Assays

Pathogenicity assays were performed on mature ‘Fuji’ apple fruits and 2-year-old ‘Fuji’ apple twigs. Apple fruits were surface-sterilized with 75% ethanol, pricked with a sterile punch to create wounds, and inoculated with 5 mm agar blocks of the tested strains. Apple twigs were surface-sterilized, cut into 10 cm segments, and wounded with a sterile knife, then inoculated with the same agar plugs. The inoculated fruits and twigs were incubated at 25 °C. The lesion length was measured at 7 dpi, and the pathogenicity was evaluated. Lesion expansion on apple twigs was quantified by measuring the longitudinal lengths of necrotic lesions. The longitudinal length of each necrotic lesion was measured separately to compare the pathogenicity levels of different strains [20]. The experiment was designed with three biological replicates, and each replicate was measured ten times.

The following equation was used: Reduction rate of lesion length (%) = [(Lesion length of wild type − Lesion length of mutant)/Lesion length of wild type] × 100%.

### 2.9. Statistical Analysis

All experimental data were analyzed using SPSS 26.0 software. The significant differences between treatments were tested by Fisher’s least significant difference (LSD) test at the *p* < 0.05 level. Graphs were plotted with Origin 2021 software.

## 3. Results

### 3.1. Bioinformatical Characterizations of VmGAL4 in V. mali

**Figure 1:**
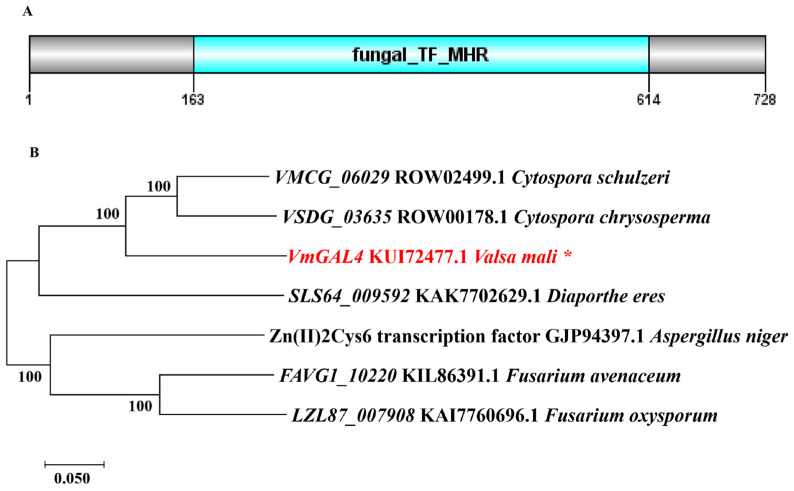
Domain prediction and phylogenetic tree construction of VmGAL4 protein. (**A**) The VmGAL4 protein consists of 728 amino acids, with a fungal_TF_MHR domain located from 163 to 614 aa. (**B**) Phylogenetic tree construction of *VmGAL4*. A phylogenetic tree was constructed by the neighbor-joining (NJ) method with MEGA 7.0 software, and the bootstrap value was set to 1000 replicates. Red fonts and asterisks indicate the target gene *VmGAL4* in this study.

The *VmGAL4* (KUI72477.1) is a single copy on chromosome 8. It has a full length of 2187 bp and encodes a protein of 728 amino acids, which contains one domain, fungal_TF_MHR, spanning residues 163 to 614 aa. These results demonstrate that the VmGAL4 protein belongs to the classic zinc finger transcription factor family and possesses the characteristic Zn_2_Cys_6_ domain (Figure 1A). Phylogenetic tree analysis revealed that the VmGAL4 protein of *Valsa mali* shared the closest phylogenetic relationship with homologs from *Cytospora schulzeri* and *Cytospora chrysosperma* (Figure 1B).

### 3.2. Acquisition of VmGAL4 Deletion Mutants and Complemented Strains

**Figure 2:**
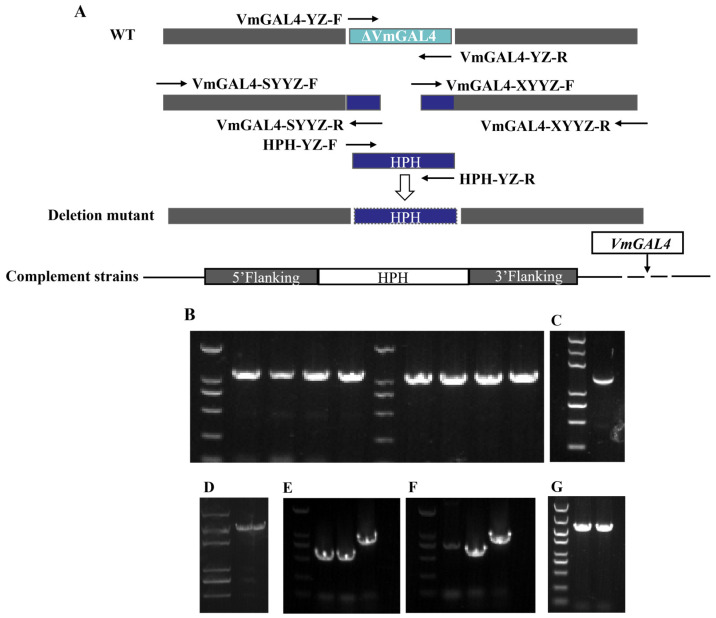
*VmGAL4* gene replacement and complementarity. (**A**) The construction strategies for deletion mutant and complemented strains. (**B**) Amplification of upstream and downstream fragments of *VmGAL4*. (**C**) Amplification of *hph*. (**D**) Double-joint PCR. (**E**) PCR screening and confirmation of the *VmGAL4* single deletion mutant using the four primer pairs as described. (**F**) PCR screening and confirmation of the *VmSom1* and *VmGAL4* double deletion mutant using the four primer pairs as described. (**G**) Obtainment of complemented strains.

The construction strategies for the deletion mutant and complemented strains are shown in Figure 2A. A total of 86 knockout transformants were obtained in this experiment. After genomic DNA extraction, four pairs of primers listed in Table S1 were used for PCR identification. Finally, one positive knockout transformant was successfully screened and designated as Δ*VmGAL4* (Figure 2B). For the complementation assay, 112 transformants were generated and screened by PCR with one pair of primers, as shown in Table S1. Two positive complemented strains, Δ*VmGAL4*-C and Δ*VmGAL4*-C-1, were obtained. The two complemented strains had identical phenotypes, so only data for Δ*VmGAL4*-C are presented.

### 3.3. VmGAL4 Regulates Vegetative Growth of V. mali

**Figure 3:**
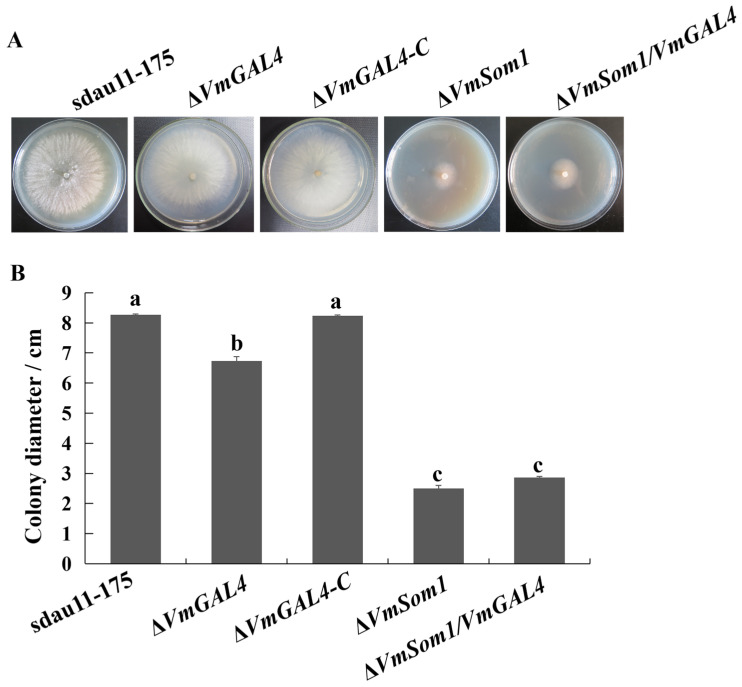
Effects of Δ*VmGAL4* on mycelial growth in *Valsa mali*. (**A**) Colonial phenotypes of strains cultured on PDA for 3 d. (**B**) Statistical results of colony diameters after 3 d of incubation. Values are presented as mean and standard deviation based on three biological replicates. Statistical analyses were carried out via Fisher’s least significant difference (LSD) test using SPSS. Different lowercase letters above bars indicate significant differences between groups (*p* < 0.05). Identical letters denote no significant difference.

Phenotypic analysis of mycelial growth on PDA medium showed that, compared to the wild-type strain sdau11-175, the colony diameter of Δ*VmGAL4* was significantly reduced, with a growth rate decreased by approximately 17.1%; additionally, the colony color was not significantly changed. The growth rate of the complementation strain Δ*VmGAL4*-C was restored to the wild-type level (Figure 3), indicating that *VmGAL4* is essential for the normal vegetative growth of *V. mali*. Compared with the Δ*VmSom1* mutant, the growth rate of the complementary strain Δ*VmSom1*/*VmGAL4* was significantly increased, yet it failed to restore to the wild-type level.

### 3.4. VmGAL4 Positively Regulates Pycnidia Production and Conidial Germination of V. mali

**Figure 4:**
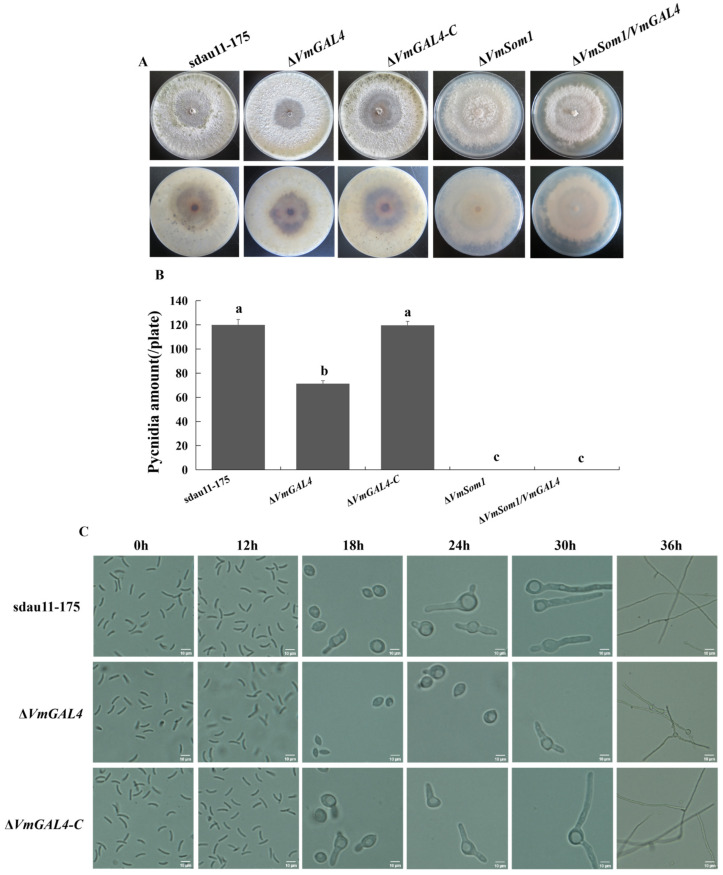
Effects of Δ*VmGAL4* on pycnidia production and conidial germination of *V. mali*. (**A**) Growth of pycnidia in sdau11-175, Δ*VmGAL4*, Δ*VmGAL4-C,* Δ*VmSom1*, and Δ*VmSom1*/*VmGAL4* on PDA for 20 d. (**B**) The number of pycnidia per unit colony area was statistically counted among different strains. Data from three replicates are analyzed using the protected Fisher’s least significant difference (LSD) test. Different lowercase letters above bars indicate significant differences between groups (*p* < 0.05), identical letters denote no significant difference. (**C**) Coating of spore suspensions from different strains onto PDA media at 25 °C for 36 h. Bars = 10 μm.

Pycnidia production assay showed that the number of pycnidia in the unit colony area of Δ*VmGAL4* was significantly reduced by about 35.9% compared with the wild-type strain (Figure 4A). To clarify the regulatory role of *VmGAL4* in conidial germination, conidial suspensions were harvested and the germination percentage was quantified via the hanging-drop incubation assay. Microscopic inspection at 18 h post-inoculation (hpi) revealed evident swelling of spores, which transformed into ellipsoid or spherical morphologies for all tested strains. At 24 hpi, wild-type conidia normally initiate germination, generating one or two germ tubes originating from opposite cell poles; meanwhile, the resultant vegetative hyphae exhibited superior elongation compared with those derived from the Δ*VmGAL4*. Despite the retarded early growth, spores of the Δ*VmGAL4* can ultimately differentiate into fully developed mature hyphae (Figure 4C). This result indicates that the Δ*VmGAL4* slows conidial germination.

Notably, both Δ*VmSom1* and Δ*VmSom1*/*VmGAL4* mutants showed severe defects in conidial production, and no pycnidia were produced on PDA medium, indicating that the deletion of *VmGAL4* cannot compensate for the conidiation defect caused by *VmSom1* deletion.

### 3.5. VmGAL4 Is Involved in Osmotic Stress and Cell Wall Integrity

**Figure 5:**
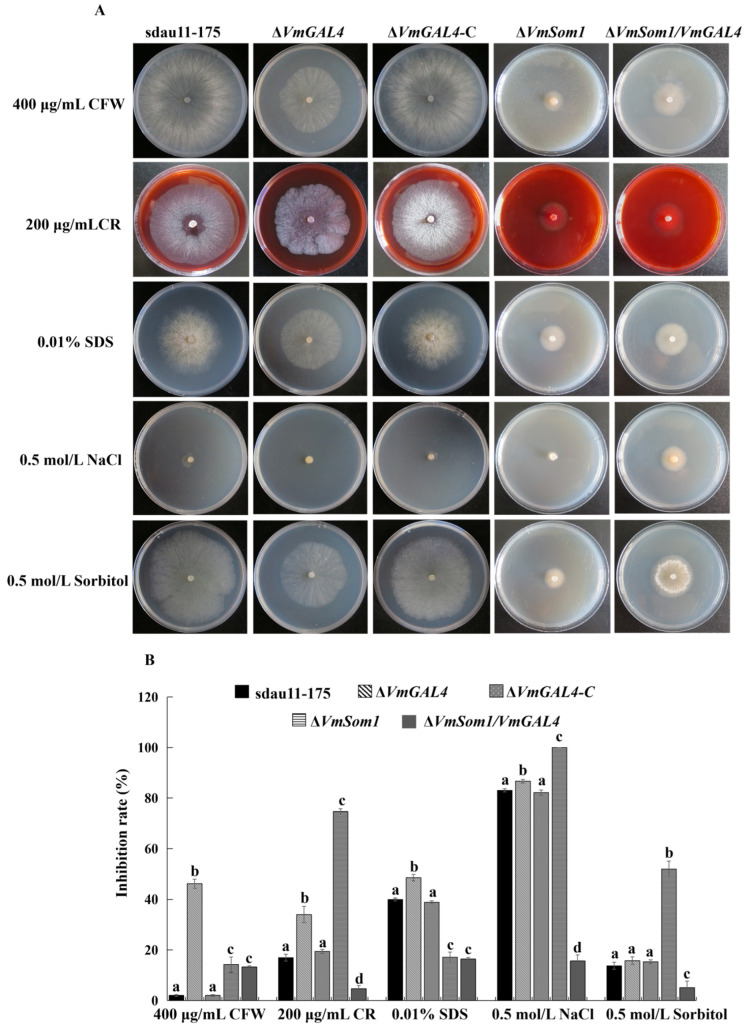
Effects of Δ*VmGAL4* on stress responses in *V. mali*. (**A**) The sdau11-175, Δ*VmGAL4*, Δ*VmGAL4-C,* Δ*VmSom1*, and Δ*VmSom1*/*VmGAL4* are inoculated on PDA supplemented with CFW, Congo red, SDS, NaCl, and sorbitol. Colony inhibition rates were calculated and fungal colonies were photographed after 3 d of cultivation. (**B**) Values are presented as mean and standard deviation based on three biological replicates. Statistical analyses were carried out via Fisher’s least significant difference (LSD) test using SPSS. Different lowercase letters above bars indicate significant differences between groups (*p* < 0.05), identical letters denote no significant difference.

In cell wall integrity assays, compared with the wild-type strain sdau11-175, the Δ*VmGAL4* exhibited markedly elevated colony inhibition rates of approximately 93.2%, 50.1% and 14.0% on PDA supplemented with 400 μg/mL Calcofluor White, 200 μg/mL Congo Red and 0.01% SDS, respectively, and the complementary strain restored growth to the wild-type phenotype (Figure 5A). For osmotic stress tests, the colony inhibition rate of Δ*VmGAL4* increased significantly by 7.5% relative to sdau11-175 on medium containing 0.5 mol/L NaCl, whereas no obvious growth difference was observed on PDA with 0.5 mol/L sorbitol (Figure 5A). These results show that *VmGAL4* regulates *V. mali*’s response to osmotic stress and maintenance of cell wall integrity.

Compared with the Δ*VmSom1*, the Δ*VmSom1*/*VmGAL4* exhibited markedly reduced sensitivity to 200 μg/mL Congo Red, 0.5 mol/L NaCl and 0.5 M sorbitol, with its sensitivity even lower than that of the wild-type. These results indicate that the deletion of gene *VmGAL4* can compensate for the deficiency of gene *VmSom1* in the medium containing Congo red, NaCl, and sorbitol.

### 3.6. VmGAL4 Negatively Regulates the Utilization of Carbon and Nitrogen Sources in V. mali

To evaluate the function of the *VmGAL4* gene in carbon source utilization, all tested strains were cultured on Czapek’s medium supplemented with distinct carbon and nitrogen sources. An equivalent concentration (30 g/L) of glucose, maltose, lactose, fructose, sucrose, and no carbon was used to formulate culture media containing distinct carbon sources. An equivalent concentration (3 g/L) of peptone, (NH_4_)_2_SO_4_, NaNO_3_, urea, and no nitrogen was used to formulate culture media containing distinct nitrogen sources.

**Figure 6:**
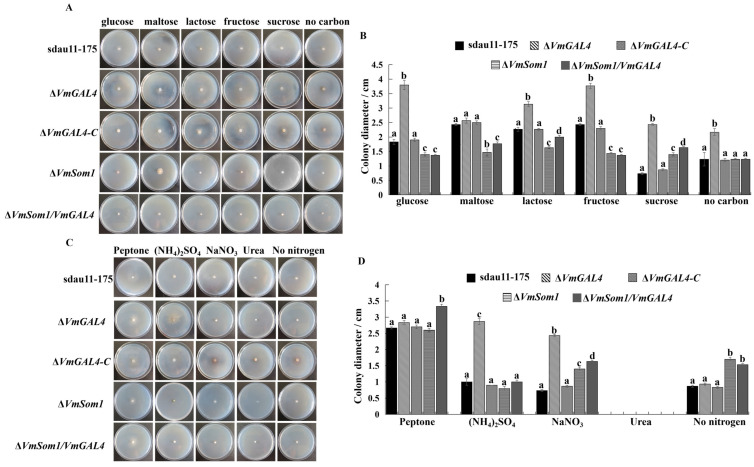
Effect of carbon and nitrogen sources on hyphal growth. (**A**) All fungal strains were cultured on Czapek’s medium supplemented with various carbon sources. Glucose, maltose, lactose, fructose and sucrose were added at the equal amounts separately to formulate media containing varied carbon nutrients. (**B**) Values are presented as mean and standard deviation based on three biological replicates. Statistical analyses were carried out via Fisher’s least significant difference (LSD) test using SPSS. Different lowercase letters above bars indicate significant differences between groups (*p* < 0.05), identical letters denote no significant difference. (**C**) All fungal strains were cultured on Czapek’s medium supplemented with various nitrogen sources. Peptone, (NH4)_2_SO_4_, NaNO_3_, and urea were added at the equal amounts separately to formulate media containing varied nitrogen nutrients. (**D**) Values are presented as mean and standard deviation based on three biological replicates. Statistical analyses were carried out via Fisher’s least significant difference (LSD) test using SPSS. Different lowercase letters above bars indicate significant differences between groups (*p* < 0.05), identical letters denote no significant difference.

The results showed that the Δ*VmGAL4* exhibited faster mycelial growth compared with the wild-type strain when cultured on media supplemented with glucose, lactose, fructose, sucrose, and carbon-free medium, respectively. On maltose medium, there was no significant difference in growth rate between the Δ*VmGAL4* and the wild-type strain. In no-carbon medium, the mycelia of sdau11-175, Δ*VmGAL4*, and Δ*VmGAL4*-C were all relatively sparse. When (NH_4_)_2_SO_4_ and NaNO_3_ were supplied as the sole nitrogen source, Δ*VmGAL4* grew more rapidly than sdau11-175. However, on nitrogen-free medium, urea, and peptone, there was no significant difference between Δ*VmGAL4* and sdau11-175. All three strains grew fastest and most vigorously on peptone medium but produced sparse mycelia on nitrogen-free medium (Figure 6A). These results indicate that *VmGAL4* negatively regulates the utilization of carbon and nitrogen sources.

Compared with Δ*VmSom1*, the Δ*VmSom1*/*VmGAL4* showed significantly increased mycelial growth rate on media supplemented with maltose, lactose, sucrose, peptone, and NaNO_3,_ respectively.

### 3.7. VmGAL4 Is Involved in the Pathogenicity of Valsa mali

**Figure 7:**
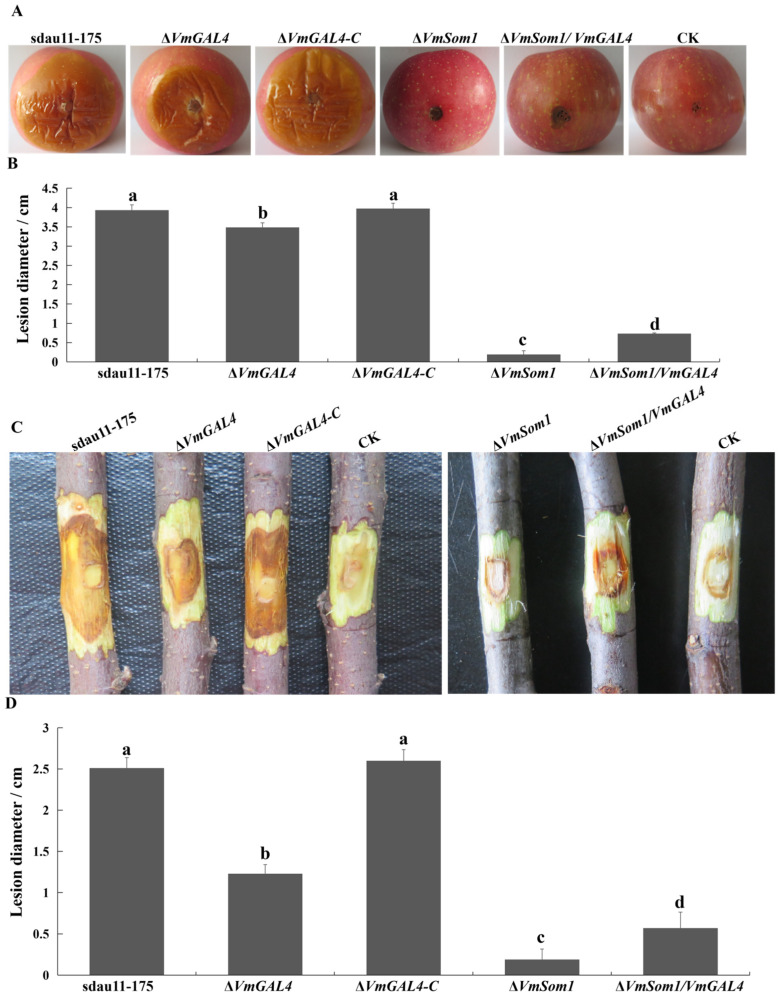
Effects of *VmGAL4* on pathogenicity in *V. mali*. (**A**) Apple fruits were surface-sterilized with 75% ethanol, pricked with a sterile punch to create wounds, and inoculated with 5 mm agar blocks of the tested strains. (**B**) The lesion length measured at 7 days post-inoculation (dpi). The experiments are repeated three times. Different lowercase letters above bars indicate significant differences between groups (*p* < 0.05), identical letters denote no significant difference. (**C**) Apple twigs were surface-sterilized with 75% ethanol, pricked with a sterile punch to create wounds, and inoculated with 5 mm agar blocks of the tested strains. (**D**) The lesion length measured at 7 dpi. The experiments are repeated three times. Different lowercase letters above bars indicate significant differences between groups (*p* < 0.05), identical letters denote no significant difference.

Pathogenicity assays on apple fruits and twigs showed that compared with the wild-type strain, the lesion length of Δ*VmGAL4* on apple fruits was reduced by about 11.4%, and the lesion length on apple twigs was reduced by about 51.0%, with significantly attenuated pathogenicity. The pathogenicity of the complementation strain Δ*VmGAL4*-C was completely restored to the wild-type level (Figure 7), indicating that *VmGAL4* is essential for the full pathogenicity of *V. mali*.

Compared with Δ*VmSom1*, the Δ*VmSom1*/*VmGAL4* showed a certain degree of recovery in pathogenicity. The lesion length on apple fruits increased by about 74.0%, and the lesion length on apple twigs increased by about 66.7%, but still significantly lower than the wild-type strain (Figure 7). These results indicate that the deletion of *VmGAL4* can partially compensate for the severe pathogenicity defect caused by *VmSom1* deletion, revealing a potential interaction between *VmGAL4* and *VmSom1* in the pathogenic process of *V. mali*.

## 4. Discussion

Zinc finger transcription factors are the largest family of transcription factors in fungi and play a central role in regulating various biological processes [26]. In this study, we identified a novel zinc finger transcription factor *VmGAL4* from *V. mali*, which encodes a protein with Zn_2_Cys_6_ zinc finger structural domains. Phenotypic analysis of single and double deletion mutants revealed that *VmGAL4* is involved in the regulation of vegetative growth, asexual development, osmotic stress response, carbon and nitrogen source utilization and pathogenicity of *V. mali*, and can partially compensate for the phenotypic defects caused by *VmSom1* deletion, revealing a complex genetic interaction between *VmGAL4* and *VmSom1* in the cAMP/PKA signaling pathway-mediated regulatory network.

Vegetative growth is the basis of fungal survival and reproduction, and transcription factors are key regulators of fungal growth [6]. Our results showed that the deletion of *VmGAL4* led to a significant reduction in the mycelial growth rate of *V. mali*, indicating that *VmGAL4* is a positive regulator of vegetative growth, which is consistent with the functions of most zinc finger transcription factors in phytopathogenic fungi [9,10]. Notably, the Δ*VmSom1*/*VmGAL4* partially restored the growth defect caused by *VmSom1* deletion. The deletion of *VmGAL4* can relieve the inhibitory effect on fungal growth caused by the loss of *VmSom1*, which reflects the functional redundancy and regulatory balance between different transcription factors in the growth regulation of *V. mali*.

Conidial production and germination are critical for the survival, spread and host infection of phytopathogenic fungi, especially for *V. mali* [3,5]. In this study, deletion of the *VmGAL4* gene reduced the production of pycnidia and delayed conidial germination in *V*. *mali*, indicating that *VmGAL4* is a positive regulator of asexual development. Similar results were found in *Botrytis cinerea* and *P*. *digitatum*, where the deletion of zinc finger transcription factor *CRZ1* led to a significant decrease in conidial yield [11,27]. However, both Δ*VmSom1* and Δ*VmSom1*/*VmGAL4* mutants lost the ability to produce conidia, indicating that *VmSom1* is a core regulatory factor for asexual development of *V. mali* that cannot be replaced by *VmGAL4*, and the asexual development regulatory pathway mediated by *VmSom1* may be independent of the *VmGAL4* pathway.

Osmotic stress response is an important adaptive mechanism of fungi to the external environment, and the ability to adapt to osmotic stress is closely related to the pathogenicity of phytopathogenic fungi [28,29]. Our study found that *VmGAL4* regulates *V. mali*’s response to osmotic stress and maintenance of cell wall integrity. In *B. cinerea*, the Zn_2_Cys_6_-type transcription factor *BcFtg1* is also required for the maintenance of cell wall integrity [30]. In *V. dahliae*, deletion of the Zn(II)_2_Cys_6_ transcription factor gene *VDAG_04814* yielded distinct stress-response phenotypes. On media supplemented with H_2_O_2_, NaCl and KCl, the growth of Δ*VDAG_04814* was suppressed to a significantly greater extent than the wild type. Conversely, the mutant exhibited remarkably lower growth inhibition than wild-type strains when exposed to sorbitol-induced osmotic stress. Despite unaltered growth under SDS and Congo red cell wall perturbing conditions, these differential stress responses collectively demonstrate that *VDAG_04814* participates in the regulation of osmotic adaptation and cell wall integrity in *V. dahliae* [31].

Pathogenicity of phytopathogenic fungi is regulated via a sophisticated regulatory network, encompassing the secretion of virulence factors, acquisition of nutrients from host tissues, metabolism of endogenous metabolites, and environmental signal perception [32,33,34]. Our study confirmed that *VmGAL4* is essential for the full pathogenicity of *V. mali*, and its deletion leads to a significant attenuation of pathogenicity to apple fruits and twigs. The attenuation of pathogenicity of Δ*VmGAL4* may be related to the combined effects of slow mycelial growth, reduced conidial production and germination rate, and altered nutrient utilization ability. Notably, the Δ*VmSom1/VmGAL4* partially restored the pathogenicity defect caused by *VmSom1* deletion, which is consistent with the partial recovery of growth rate. However, the pathogenicity of Δ*VmSom1/VmGAL4* is still significantly lower than that of the wild-type strain, indicating that *VmSom1* is the core regulatory factor of pathogenicity, and *VmGAL4* can only partially compensate for its function, and there may be other downstream genes involved in the *VmSom1*-mediated pathogenicity regulatory pathway.

Based on our phenotypic results and existing fungal regulatory network studies, we propose a plausible hypothesis for the interaction between *VmGAL4* and *VmSom1*. *VmSom1* functions as a downstream core effector of the cAMP/PKA signaling pathway and dominates the regulation of vegetative growth, asexual development, and pathogenicity in *V. mali*. *VmGAL4* possesses partial functional redundancy with *VmSom1* in regulating vegetative growth and pathogenicity. Such a hierarchical regulatory mode consisting of transcription factors is conserved in fungal cAMP/PKA signaling networks, which ensures the accuracy and flexibility of biological regulation [35]. They may co-regulate a set of genes associated with mycelial growth and nutrient utilization, while *VmSom1* specifically controls core genes for asexual development and key virulence factors that cannot be activated by *VmGAL4*. This explains why *VmGAL4* can partially restore growth and pathogenicity defects but fails to recover conidiation deficiency in the double mutant. Collectively, our findings indicate that the cAMP/PKA pathway fine-tunes the growth, development, and pathogenicity of *V. mali* through the synergistic regulatory module of *VmSom1* and *VmGAL4*.

## 5. Conclusions

In this study, we constructed the *VmGAL4* single deletion mutant and *VmSom1/VmGAL4* double deletion mutant of *V. mali* and systematically analyzed their phenotypic characteristics. The results showed that *VmGAL4* plays critical roles in vegetative growth, conidial production and germination, pathogenicity, osmotic stress response and carbon/nitrogen source utilization of *V. mali*. In addition, *VmGAL4* can partially compensate for the defects in growth rate, osmotic stress response and pathogenicity caused by *VmSom1* deletion, but cannot restore the conidiation defect caused by *VmSom1* deletion. Our study reveals the functional characteristics of *VmGAL4* and its genetic interaction with *VmSom1*, providing new insights into the molecular regulatory mechanism of *V. mali* and a theoretical basis for the sustainable control of apple canker.

## Data Availability

All the data that support the findings of this study are available in the paper and its Supplementary Material published online.

## Supplementary Materials

The following supporting information can be downloaded at https://www.mdpi.com/article/10.3390/jof12070511/s1: Table S1: Primers used in this study.

## Abbreviations

The following abbreviations are used in this manuscript:

CR	Congo Red
CFW	Fluorescent Brightener
SDS	Sodium Dodecyl Sulfate
