# Supplementary material for: Transcription Factor VmGAL4 Governs Vegetative Growth, Development, and Virulence in Valsa mali
Source: J Fungi (Basel). 2026 Jul 12;12(7):511. doi: 10.3390/jof12070511 (PMC13412484; doi:10.3390/jof12070511)
Supplement: Supplementary file 1 [file jof-12-00511-s001.zip › jof-4393707-supplementary.pdf]

Table S1. Primers used in this study

Fragments	Primers	Sequences (5'-3')
Upstream segments	VmGAL4-SY-F	GCTGTGAAGAATGGCGTAT
	VmGAL4-SY-R	attcattgttgacctccactTCAGGAGGTATCTCGTTGG
Downstream segments	VmGAL4-XY-F	gggcaaaggaatagagtaCTGTTCAACGGCAAATCG
	VmGAL4-XY-R	CACCGCAGCCTACACCAT
HPH segments	HPH-F	AGTGGAGGTCAACAATGAAT
	HPH-R	TCTACTCTATTCCTTTGCCC
PCR Validation L1	VmGAL4-SYYZ-F	AGGCGGATATGTAGGACG
	VmGAL4-SYYZ-R	CCATAAGGTCAATCGCTGT
PCR Validation L2	VmGAL4-XYYZ-F	TTGCCGTATTAGATTAGGTCG
	VmGAL4-XYYZ-R	CCACGCTTTCTCCGCTTA
PCR Validation HPH	VmGAL4-YZ-F	TATTAGCAGACAGGAACGAGGAC
	VmGAL4-YZ-R	CTTCTGCGGGCGATTTGTGTA
VmGAL4 complementation	HPH-YZ-F	actcactatagggcgaattgggtactcaaattggttACCTTATGGT CCTGTTTCC
	HPH-YZ-R	caccaccccggtgaacagctcctcgcccttgctcacTATTTGCGTT CTTTCTCACT
Detection complementation strain	VmGAL4-HB-F	ATGGTGAGCAAGGGCGAGGA
	VmGAL4-HB-R	ACTAGTTTACTTGTACAGCTCGTCCATGC
